# Investigating white matter perfusion using optimal sampling strategy arterial spin labeling at 7 Tesla

**DOI:** 10.1002/mrm.25333

**Published:** 2014-06-20

**Authors:** Alexander G Gardener, Peter Jezzard

**Affiliations:** FMRIB Centre, Nuffield Department of Clinical Neurosciences, University of Oxford, John Radcliffe HospitalOxford, United Kingdom

**Keywords:** ASL, white matter, CBF, perfusion, ultra-high field

## Abstract

**Purpose:**

Cerebral blood flow (CBF) is an informative physiological marker for tissue health. Arterial spin labeling (ASL) is a noninvasive MRI method of measuring this parameter, but it has proven difficult to measure white matter (WM) CBF due to low intrinsic contrast-to-noise ratio compared with gray matter (GM). Here we combine ultra-high field and optimal sampling strategy (OSS) ASL to investigate WM CBF in reasonable scan times.

**Methods:**

A FAIR-based ASL sequence at 7T was combined with a real-time-feedback OSS technique, to iteratively improve post-label image acquisition times (TIs) on a tissue- and subject-specific basis to obtain WM CBF quantification.

**Results:**

It was found 77% of WM voxels gave a reasonable CBF fit. Averaged WM CBF for these voxels was found to be 16.3 ± 1.5 mL/100 g/min (discarding partial-volumed voxels). The generated TI schedule was also significantly different when altering the OSS weighted-tissue-mask, favoring longer TIs in WM.

**Conclusion:**

WM CBF could be reasonably quantified in over 75% of identified voxels, from a total preparation and scan time of 15 min. OSS results suggest longer TIs should be used versus general GM ASL settings; this may become more important in WM disease studies. Magn Reson Med 73:2243–2248, 2015. © 2014 The Authors. Magnetic Resonance in Medicine published by Wiley Periodicals, Inc. on behalf of International Society for Magnetic Resonance in Medicine. This is an open access article under the terms of the Creative Commons Attribution License, which permits use, distribution and reproduction in any medium, provided the original work is properly cited.

## INTRODUCTION

MRI arterial spin labeling (ASL) is a noninvasive method of labeling arterial blood to investigate cerebral blood flow (CBF) 1–3. Tissue perfusion is an important physiological marker and ASL has applications in studying function and pathology. Following the labeling RF pulse (pulsed ASL, PASL) or labeling period (continuous ASL, CASL), there is a time delay (TI) before acquisition of images superior to labeled blood vessels, to allow perfusion into the tissue. An otherwise-matched control image (without labeling) is acquired on alternate acquisitions. On comparing the labeled (tag) and nonlabeled (control) images the difference is found to depend on voxel perfusion. Using calibration scans, acquiring data at multiple TIs and postprocess fitting to a suitable model, voxel-by-voxel quantification of CBF and blood arterial transit time (ATT) can be achieved 4. However, most ASL studies focus on the investigation of gray matter (GM) tissue 5–7.

The study of white matter (WM) CBF by ASL has proven challenging, mainly due to low intrinsic difference signal (ΔM) between control and tag images, which limits the contrast-to-noise ratio (CNR). From literature the WM ΔM signal is reported to be 2–4 times smaller than GM ΔM, with arterial arrival times also longer, leading to increased relaxation (and signal loss) of labeled blood before arrival and perfusion into WM tissue 8. There are also concerns that partial voluming of GM within tissue voxels leads to over-estimation of the WM CBF. Nevertheless, there is increasing interest in using ASL for white matter disease studies and so robust quantification methods are highly desirable, for instance in WM leukoaraiosis and Alzheimer’s disease 9–11.

Recent studies have investigated developments in labeling sequences, such as the pseudo-Continuous ASL (pCASL) technique 12. In one study, pCASL was used to investigate the number of acquisitions of single postlabel delay time (PLD) images and effect on WM CNR 13; in another, multiple-PLD pCASL acquisition was combined with fine detail ROI analysis 14. Furthermore, PASL acquisition, combined with single-voxel spectroscopic-like localization, has been suggested to overcome CNR and arrival time limitations 15. With the availability of ultra-high field (UHF) human scanners an alternative approach is available, to take advantage of higher SNR and longer T_1_ blood relaxation times at these field strengths, which benefit ASL 16,17. Coupled with an optimal sampling strategy (OSS) approach, to optimize the selection of TI times for WM CBF measurement using real-time feedback 18, the aim of this study was to demonstrate WM CBF quantification and acquire data in a timeframe suitable for future multi-parameter white matter disease investigations.

## METHODS

Scanning was performed using a 7 Tesla (T) Siemens whole-body scanner (Siemens Healthcare, Erlangen, Germany), equipped with a combined head-only 32-channel receive and single-channel transmit coil (Nova Medical, Wilmington, MA). The physical dimensions of this coil limited the transmit RF coverage to the region superior to the subject’s neck, with an inside diameter of 290 mm and length 280 mm; the receive coil had diameter 180 mm and height 240 mm. Due to ultra-high field B_0_/B_1_ profile and efficiency issues 16,19, a multiple-TI Flow-sensitive Alternating Inversion Recovery (FAIR) Quantitative Imaging of Perfusion Using a Single Subtraction (QUIPSS2) PASL sequence was used for the study 3,17,20. In FAIR a nonselective inversion pulse is centered on the image volume as the tag condition; a selective inversion covering the image slab is the control, so inflowing blood is not inverted. Subtraction of the two leaves a perfusion-weighted signal difference, as static tissue in the image is the same in both. Frequency-Offset Corrected Inversion (FOCI) label pulses, and Shinnar-Le Roux (SLR)-optimized saturation pulses (for image pre- and postlabel saturation and QUIPSS2), were used in this implementation 21–23. Pulse efficiencies were assessed using a Polydimethylsiloxane (PDMS) oil phantom (T_1_ of 1400 ms at 7T). These were found to be better than 95% of desired flip-angle across spatial profile, at up to ±10 cm from B_0_ isocenter. The sharpness of the inversion and saturation pulse edges was less than 1 voxel (3 mm) width for ramp, for up to 20 cm pulse thickness (nonselective inversion thickness).

For this study an OSS approach was used with FAIR, to iteratively improve TI_2_ values as the scan progressed using real-time feedback, optimizing delay time choice 18,24. In OSS-ASL, N pairs of control-tag images (where N equals number of TIs) are acquired, with an improved schedule of N TI_2_s generated from included voxels 24. This is fed back to the scanner control, updating sequence timings, and the new TI_2_ set acquired. This process repeats to generate an ever-improving TI_2_ set. All generation and feedback functions ran on the image-processing pipeline on the reconstruction computer, running in the background whilst data were acquired as per normal operation.

**Figure 1:**
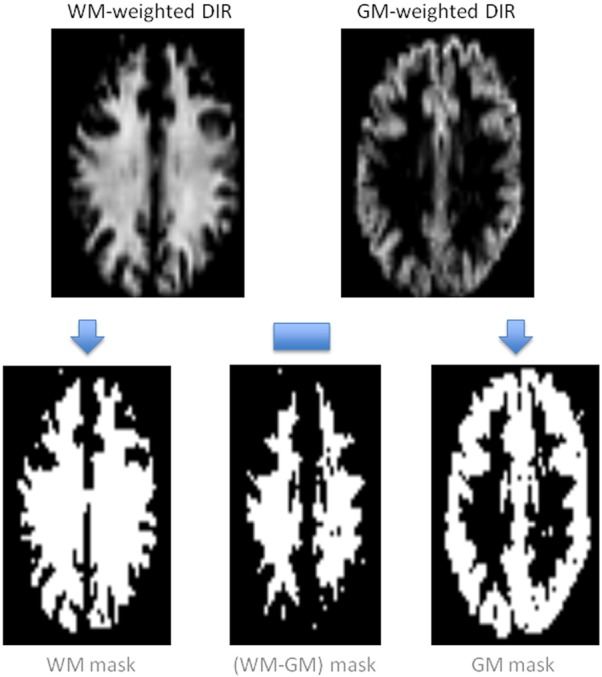
Schematic showing the acquired tissue-weighted DIR images, their thresholding into tissue masks, and subsequent (WM-GM) voxel subtraction to remove partial-volumed voxels. These were then used in OSS TI_2_ generation on the scanner.

Eight healthy adult male subjects were scanned (ages 33 ± 5 years), giving informed consent under an institutional development protocol. Unless noted all imaging was performed at a resolution of 3 × 3 × 5 mm^3^ with a 64^2^ matrix, with six axial slices (2 mm slice gap) acquired in ascending order. Gradient-echo EPI acquisition (6/8 partial k-space coverage) was used, with flow-crushing gradients in the slice-direction (critical velocity of 5 cm/s) to suppress unwanted large vessel signal. Double inversion recovery (DIR) images were acquired before the OSS-ASL scans to generate voxel masks for the OSS process. These were thresholded to binary masks and stored in scanner memory until used by OSS. Three approaches were used; (A) WM voxel mask (TR 30 s; TI values of 0, 4300 ms, acquire at 5400 ms); (B) GM voxel mask (TR 30 s; TI values of 0, 3560 ms, acquire at 4220 ms); and (C) a WM-GM mask constructed from the WM-mask minus overlapping GM voxels, to minimize partial volume effects. Representative DIR images and masks are presented in Figure 1. DIR timings were generated from T_1_-based simulations, and were adjusted at the scanner for the best tissue contrast 25. For FAIR scans a QUIPSS2 pulse was applied at 0.7 s postlabel inversion (TI_1_). A long TI_1_ would limit the minimum achievable TI_2_ value, whilst a shorter TI_1_ might reduce the amount of tagged blood delivered to the brain; values of 0.6 to 1.2 s have been suggested 20. Pre/postinversion saturation pulses were used to reduce potential B_1_ profile errors. TE was set as short as possible at 18 ms, including time for flow-crushers. The TR was 4 s, which gave 95% SAR at 7T; this limited the maximum number of slices. The selective inversion was wider than the imaging slab to prevent interference from poor pulse profile; effective gap between bolus edge and the inferior slice was 20 mm. The OSS TI_2_ search space was set to 0.8 s to 2.55 s, to fill the TR period and allow OSS flexibility in optimization fits for either tissue. Eight initial TI_2_ values were acquired in a block at the start of each OSS-ASL, evenly spaced every 0.25 s; for subsequent blocks OSS-generated TI_2_ values were used. For each of the OSS scans 160 images (10 blocks) were acquired, taking 11 min in total per ASL scan. An unprepared M_0_ image was acquired for CBF quantification, with a TR of 20 s but matching ASL scan parameters. With the preparation (DIR), calibration (M_0_) and tissue-weighted OSS scans, total duration per subject was approximately 45 min.

### Analysis

Data were processed off-line using FSL tools 26 and Matlab (Mathworks, Natick, MA) to prepare ΔM time-series. Motion-correction and registration to M_0_ calibration image, brain extraction and multi-channel coil correction were performed before pair-wise control-tag subtraction. The “Bayesian Inference for Arterial Spin Labeling” (BASIL) tool was used to fit ΔM data to a single-compartment ASL kinetic model (voxel-by-voxel) using all TI_2_ values from each scan 4,27. The effects of acquisition delay on the TI_2_ value for each slice and the WM and GM T_1_ relaxation times at 7T were accounted for in fitting (tissue T_1_ 1.4 and 1.9 s, respectively) 28,29. The M_0-blood_ term in the standard model was approximated from M_0-csf_, measured in the ventricles (corrected for T_2_^*^ decay and blood:water partition coefficient) 30.

Fitted parameters were investigated using ROIs generated from thresholded, brain-extracted DIR images registered to M_0_ images. Voxels found to be in GM and WM- thresholded ROIs were discarded to avoid partial volume contamination. BASIL-generated z-statistics for the CBF fit were used as a marker of goodness-of-fit to the model parameters 27,31. It was found that the third slice acquired contained the highest number of WM-voxels. For consistency this slice was used to investigate a representative ATT for labeled blood. The OSS-generated TI_2_ sets were also compared for the different tissue weightings.

## RESULTS

Table1 presents mean CBF values for tissue ROIs, averaged over all slices for subjects, then across subjects. The ‘GM’ row indicates gray matter voxels without overlapping WM voxels; “(WM–GM)” includes all voxels identified from the WM minus the GM mask; “(WM-GM) z>2” used the same base ROI, but only includes voxels with a fitted CBF z-stat greater than 2 (approximately 95% confidence in fitted value, *P* = 0.05). Across subjects this was found to be 77 ± 8% of “(WM-GM)” voxels; these are referred to as “significant (WM-GM)” voxels. The mean CBF of WM increased by 2.7 mL/100 g/min when poorly fitted voxels were excluded (second versus third row of Table1). The increase in subject 5 CBF after discarding poorly fitting voxels accounts for the increased standard deviation in the significant (WM-GM) voxels. The bottom row presents average CBF for the whole WM mask (z-stat > 2), including any overlapping GM voxels. This is significantly higher (*P* < 0.01) across all subjects than the significant (WM-GM) CBF, likely due to partial-volume GM contamination. DIR images show good demarcation between GM and WM, checked against MNI-152 library segmentations. These results emphasize the importance of voxel consideration in ROI analysis. It would be worthwhile investigating WM CBF across a wider sample set to improve the precision of these values 32. There was greater variation across subject GM CBF values, with two subjects having particularly high and low outliers. Excluding these outliers in the average GM CBF gave a similar mean but reduced standard deviation (to 50.4 ± 6.0 mL/100 g/min).

**Table 1:** Subject and Averaged CBF Values from BASIL Fits for: GM, All (WM-GM); Significant (z-stat >2) (WM-GM); and Lastly All (z-stat >2) WM Voxels, which Includes Some GM Partial-Voluming Effects

Subject	1	2	3	4	5	6	7	8	Mean CBF (mL/100 g/min)
GM CBF	43.3	33.6	51.2	48.8	69.3	57.6	58.2	43.4	50.7 ± 11.0
(WM-GM) CBF	13.2	14.0	13.9	13.6	14.5	12.8	14.2	12.2	13.6 ± 0.8
(WM-GM) z>2 CBF	16.3	15.4	16.5	16.6	19.6	15.0	16.3	14.7	16.3 ± 1.5
WM z>2 CBF	17.9	16.6	17.4	18.5	20.1	16.1	17.4	17.5	17.7 ± 1.2

**Figure 2:**
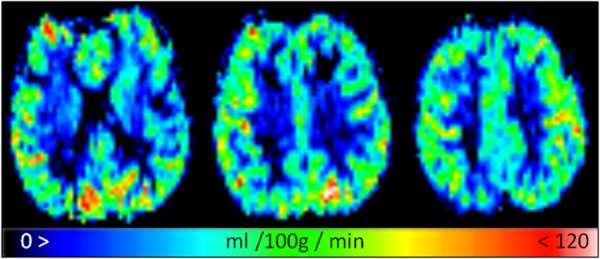
Fitted representative CBF maps for a GM-like perfusion range of 0 to 120 mL/100 g/min.

**Figure 3:**
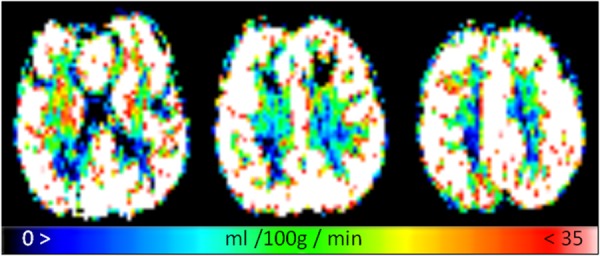
The same fitted CBF images as Figure 2, but rescaled to a more WM-like perfusion range of 0 to 35 mL/100 g/min to highlight WM tissue regions.

**Figure 4:**
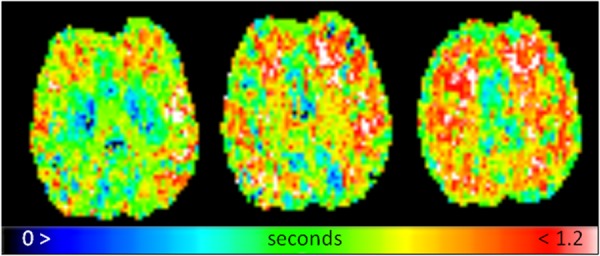
Fitted representative ATT maps, slices to match Figures 2 and 3; displayed on a range of 0 to 1.2 s.

Figure 2 presents representative CBF maps for 3 slices, and Figure 3 presents the same images but with perfusion scaled specifically to a WM CBF range for clarity (with any value above 35 mL/100 g/min shown as white). The two tissue regions exhibit fairly even CBF distributions. Figure 4 presents representative fitted ATT maps, to match Figure 2; slice 3 is the right-most image. Longer ATT values are seen in WM regions, as expected. Representative fitted ATT values are presented in the Supporting Information (Supporting Table S1, which is available online) for a single slice (matching slice across subjects). As expected, significant (WM-GM) ATT values are longer than those found in GM by an average 0.19 s, reflecting later arrival of labeled blood to WM tissue. This would be difficult to observe in a single-TI ASL experiment; it may also vary in WM-disease studies from aberrant (faster or slower) blood flow 33.

Supporting Figure S1 plots the real-time generated OSS TI_2_ distributions for the final OSS TI_2_ block, averaged across subjects for each OSS (tissue) type. It shows longer TI_2_ values are preferred for WM OSS, and longer still for (WM-GM) OSS, likely reflecting the later arrival of labeled blood. This causes a shift of the kinetic model peak; the OSS algorithm then favors a later acquisition window versus GM OSS. Using a paired two-tailed Student’s t-test there was found to be significant difference between the (WM-GM) and GM OSS TI_2_ distributions (*P* < 0.01), suggesting OSS was masking-tissue dependent and showing sensitivity to blood delivery.

## DISCUSSION

This study shows it is possible to obtain CBF and ATT values for WM at 7T using an OSS FAIR-QUIPSS2 PASL sequence, in 15 min of acquisition including preparation, calibration and 11 min of ASL scanning. Prior work had attempted to investigate WM perfusion using PASL at 3T, but it was found that WM CBF fits were poor even with 20 min of data acquisition; over 80% of voxels were not fitted or had low z-stat, suggesting uncertainty in the CBF values obtained. The combination of ultra-high field and OSS proved a powerful tool in this initial study, with over three quarters of identified WM voxels having what were considered reasonable CBF fits. The parameter values found are comparable to literature, with a significant (WM-GM) CBF value that is slightly lower than in previous studies, reflecting care in selecting ROI voxels (avoiding partial volume effects) and discarding 23% of poor CBF-fitted voxels. The ratio of GM to significant (WM-GM) CBF values was found to be 3.1:1, falling within a range from previous reports [ratios: 2.5:1 to 5:1 8,13–15,33–35]. ATT values showed a difference of approximately 0.2 s between GM and significant (WM-GM), similar to previously reported; due to specific ASL settings this difference is a more useful marker than absolute ATT value 14,33,35,36.

Using OSS gave information on suitable TI_2_ ranges for WM ASL compared with GM ASL. What may be optimal acquisition times for one tissue may not be optimal for another. There is little currently in the literature exploring this, especially at ultra-high field-strength where T_1_ values are longer. Often, protocols are based on lower-field studies and used with little modification. Furthermore, there is an argument that CBF fits from ΔM benefits from having a large set of delay times, against repeat acquisitions at a few TI_2_ values 27,37. OSS intrinsically provides such data, with subject- and tissue-specific TI_2_ values generated during acquisition. The results suggest for healthy adults a range of 1.1 to 2.3 s is suitable for WM, which is a longer than is typically used in lower field / GM-tissue studies. A range of 1.0 to 2.0 s is suggested for GM at 7T; theory suggests a peak around 1.8 s to match tissue/blood T_1_ values. This study used a relatively high number of TI_2_ values (eight) to give the OSS a large data set for its optimization. It would be worth investigating if a smaller number of TIs and acquisition blocks resulted in similar TIs and WM CBF values; this would allow a reduction in ASL scan time, to move toward a clinically useful total time (ideally less than 10 min for everything).

The resolution of images acquired in this study kept EPI acquisitions short with lower TE, and provided more signal per voxel. We assessed CNR from mean difference signal divided by background region standard deviation; at max signal (3rd/4th TI typically) the ratio of GM:WM CNR was 3.1:1; by the 8th TI this was 2.4:1, showing a greater proportional loss in GM CNR (and signal), but still remaining above WM CNR. The limit of viable CNR for longer TIs, such as for reduced or delayed perfusion disease studies, is an area of interest in future studies. Higher resolution requires additional data acquisition due to lower CNR, and results in longer TEs, introducing more BOLD signal contamination. At 7T the optimal TE for BOLD shortens to 25–30 ms from 30–50 ms at 3T, depending on region. Parallel imaging was not used in this study, as it provided no reduction in TE and complicated real-time processing; however, it may reduce TE in a high-resolution study. One advantage of high-resolution acquisition would be to further improve ROI selection, limiting partial volume contamination and allowing regional or structural ROI analysis.

The potential of OSS-ASL in studying WM perfusion in ageing and/or disease is an area of particular interest. Such studies may benefit from OSS adapting to unusual blood dynamics in subjects, with optimal TI_2_s varying from healthy subjects; this information may not be known before scanning. Using a wide TI_2_ search space and allowing OSS sufficient optimization runs should provide subject- and/or pathological-optimized TI_2_ values. It is worth noting that OSS ΔM images retain all ASL perfusion information compared with standard ASL, whilst offering additional information for little additional cost. Postprocessing is similar to standard ASL analysis, substituting individual TI_2_ values for each ΔM image rather than repeated blocks of TI_2_ values.

The current lack of a scanner body-transmit coil, or a dedicated neck-labeling coil, prevented implementation of a higher CNR OSS-pCASL technique in this study; the latter coil is under development. This would potentially allow another method of obtaining high-resolution ASL imaging; alternatively, pCASL could reduce the number of acquisitions needed per scan, making the shorter pCASL acquisition more clinically useful, though this risks reducing the optimization power of OSS if too few blocks are generated. A problem that remains with ultra-high field systems is SAR; this is not limited to ASL. Parallel transmit methods may offer future solutions to this issue, as well as offering improved label pulses, or novel methods of vessel suppression.
