# Supplementary material for: Investigating white matter perfusion using optimal sampling strategy arterial spin labeling at 7 Tesla
Source: Magn Reson Med. 2014 Jun 20;73(6):2243–8. doi: 10.1002/mrm.25333 (PMC4657501; doi:10.1002/mrm.25333)
Supplement: Supplementary file 1 — Supplementary Information [file mrm0073-2243-sd1.docx]

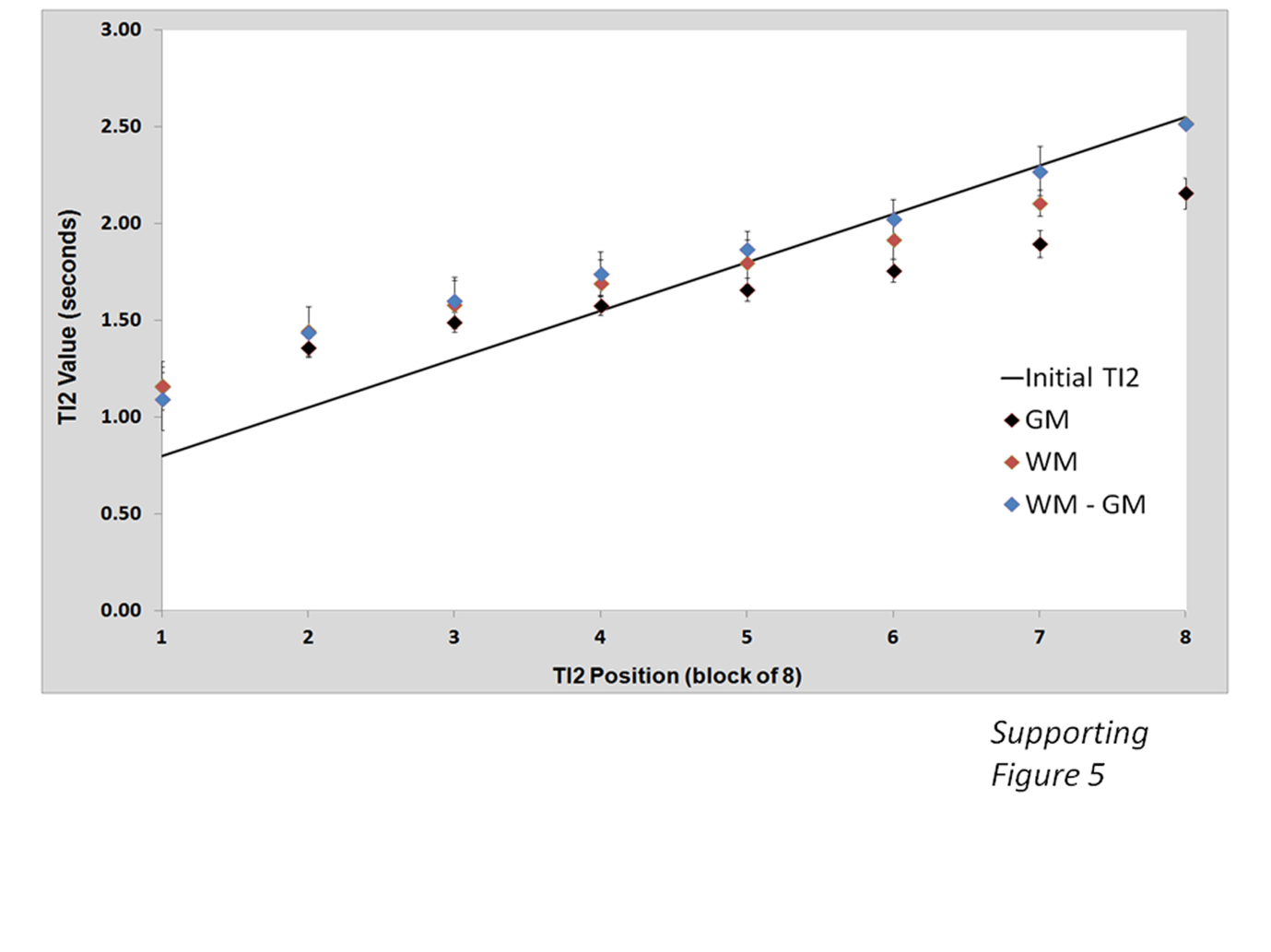


Supporting Figure 5: The end set of eight OSS-generated TI_2_ values, for different tissue-mask primers, averaged across the group. These should be the optimal TI_2_ schedule for the voxels within their respective masks, since they account for all previous ΔM data in that scan. The increase in preferred TI_2_ values with mask type [GM < contaminated WM < (WM-GM)] can be seen.

| *Subject* | *1* | *2* | *3* | *4* | *5* | *6* | *7* | *8* | Mean ATT  (seconds) |
| --- | --- | --- | --- | --- | --- | --- | --- | --- | --- |
| GM  ATT | 0.63 | 0.74 | 0.86 | 0.74 | 0.83 | 0.87 | 0.79 | 0.64 | 0.77 ± 0.09 |
| (WM-GM)  ATT | 0.83 | 0.86 | 0.98 | 0.95 | 0.92 | 0.97 | 0.95 | 0.86 | 0.92 ± 0.06 |
| (WM-GM)  z>2 ATT | 0.85 | 0.92 | 1.03 | 1.00 | 0.99 | 1.01 | 0.96 | 0.88 | 0.96 ± 0.06 |

Supporting Table 2: Subject and averaged arterial transit time (ATT) for labeled blood from BASIL fits, for: GM; all (WM-GM); and significant (z-stat >2) (WM-GM) voxels. These are taken from the 3rd slice acquired in each subject, which generally had most WM voxels.
